# Supplementary material for: Mutation load dynamics during environmentally-driven range shifts
Source: PLoS Genet. 2018 Sep 28;14(9):e1007450. doi: 10.1371/journal.pgen.1007450 (PMC6179293; doi:10.1371/journal.pgen.1007450)
Supplement: S1 Table — Absolute fitness loss and mutation fixations during expansion per 1-D simulation scenario, averaged over 10 replicate simulations. Cases indicated with a * go extinct before the expansion completes. T indicates the number of generations between which the population moves over the landscape and v is the speed of spread (inverse of T, as defined in the Methods). (PDF) [file pgen.1007450.s001.pdf]

Table S1. Fitness loss and mutation accumulation across scenarios. Absolute fitness loss and mutation fixations during expansion per 1-D simulation scenario, averaged over 10 replicate simulations. Cases indicated with a * go extinct before the expansion completes. *T* indicates the number of generations between which the population moves over the landscape and *v* is the speed of spread (inverse of *T*, as defined in the Methods).

Selection	Scenario	T	*v*	h	Expansion time (generations)	Mean absolute fitness loss	Mean fitness loss per generation	Mean fitness loss per deme	Avg. no. of deleterious mutations fixed	Avg. no. of beneficial mutations fixed
Soft	Expansion	3.87 (realized)	0.258	0.5	1200	0.3269	2.72e-4	1.15e-3	144.3	10.9
		3.91 (realized)	0.256	0	1300	0.4647	3.57e-4	1.65e-3	174.3	8.8
	Shift	5	0.2	0.5	1500	0.3658	2.44e-4	1.28e-3	159.7	15.6
				0	1500	0.5030	3.35e-4	1.77e-3	191.3	12.7
		10	0.1	0.5	3000	0.5119	1.71e-4	1.74e-3	219.1	28
				0	3000	0.6181	2.06e-4	2.10e-3	259.3	25.9
		20	0.05	0.5	>5000	0.5551	1.11e-4	2.18e-3	272.1	52
				0	>5000	0.6808	1.36e-4	2.67e-3	321.5	46.6
		50	0.02	0.5	>5000	0.2081	4.16e-5	1.98e-3	116.2	64.5
				0	>5000	0.2974	5.95e-5	2.83e-3	132.7	52.8
Hard	Expansion	5.66 (realized)	0.177	0.5	1700	0.3962	1.65e-4	1.33e-3	154	16.9
		20.6 (realized)	0.049	0	>5000	0.5107	1.02e-4	2.11e-3	236.1	59.5
	Shift	5	0.2	0.5	1100*	0.2454	2.23e-4	1.11e-3	119	14
				0	300*	0.2268	7.56e-4	3.60e-3	104.2	2.67
		10	0.1	0.5	2500*	0.4293	1.71e-4	1.68e-3	174.4	25
				0	1600*	0.4207	2.63e-4	2.57e-3	168.2	17
		20	0.05	0.5	3400*	0.4815	1.41e-4	2.75e-3	271	42
				0	2300*	0.4994	2.17e-4	4.16e-3	231	18
		50	0.02	0.5	>5000	0.2944	5.89e-5	2.80e-3	150.1	65.4
				0	>5000	0.3888	7.78e-5	3.70e-3	184.6	57.7
