# Supplementary material for: Mutation load dynamics during environmentally-driven range shifts
Source: PLoS Genet. 2018 Sep 28;14(9):e1007450. doi: 10.1371/journal.pgen.1007450 (PMC6179293; doi:10.1371/journal.pgen.1007450)
Supplement: S2 Table — All parameter combinations simulated in the current study to ensure reproducibility of the results. Software code can be downloaded from https://github.com/kjgilbert/ExpLoad. Parameters written in italics within parentheses are the exact software input names used by the simulation. 10 replicate simulations were run with data saved every 100 generations. (PDF) [file pgen.1007450.s002.pdf]

Table S2. Simulation parameters. All parameter combinations simulated in the current study to ensure reproducibility of the results. Software code can be downloaded from https://github.com/kjgilbert/ExpLoad. Parameters written in italics within parentheses are the exact software input names used by the simulation. 10 replicate simulations were run with data saved every 100 generations.

**Parameter**	**Setting(s) Used**
Genome-wide mutation rate (*mu*)	0.1
Migration rate (*m*)	0.1
Selection (*selectionMode*)	Soft (0), Hard (1)
Selection coefficient (*s*)	+/-0.005, exponential DFE with mean = +/-0.005
Dominance, *h* (set with line 301 in Individual.cpp)	0.5, 0.3, 0.0, *h-s* trade-off (see Methods)
Proportion deleterious loci (*phi*)	0.9
Scenario (*expansionMode*)	Linear (0)
Demographic scenario (*expansionModeKim*)	Expansion (0), Shift (2)
T, number of generations between each step of the shift (*theta*)	NA (free expansion), 5, 10, 15, 20, 25, 30, 40, 50, 60, 84
Generations after burn-in (*gens.to.run*)	4000
Landscape width (*m1*)	1, 5
Landscape length (*m2*)	300
Ancestral population size (for burn-in; *anc_pop_size*)	10000
Ancestral burn-in generation time (*burnin_time*)	10
Starting population size (*starting_demes*)	5
Starting population burn-in on landscape (*expansion_start*)	5000
Range shift population size (*niche_width*)	5
Carrying capacity (*capacity*)	20, 100, 500
