# Supplementary material for: Mutation load dynamics during environmentally-driven range shifts
Source: PLoS Genet. 2018 Sep 28;14(9):e1007450. doi: 10.1371/journal.pgen.1007450 (PMC6179293; doi:10.1371/journal.pgen.1007450)

Figure S1. **Mutation fixation through time.** Fixation of deleterious (A, C, E, G) and beneficial (B, D, F, H) mutations at the expanding range front, under soft and hard selection on a 1-dimensional landscape. Vertical lines indicate when the landscape has been crossed and expansion is complete; extinction has occurred for lines that end abruptly. Shaded area indicates two standard errors over 10 replicates.

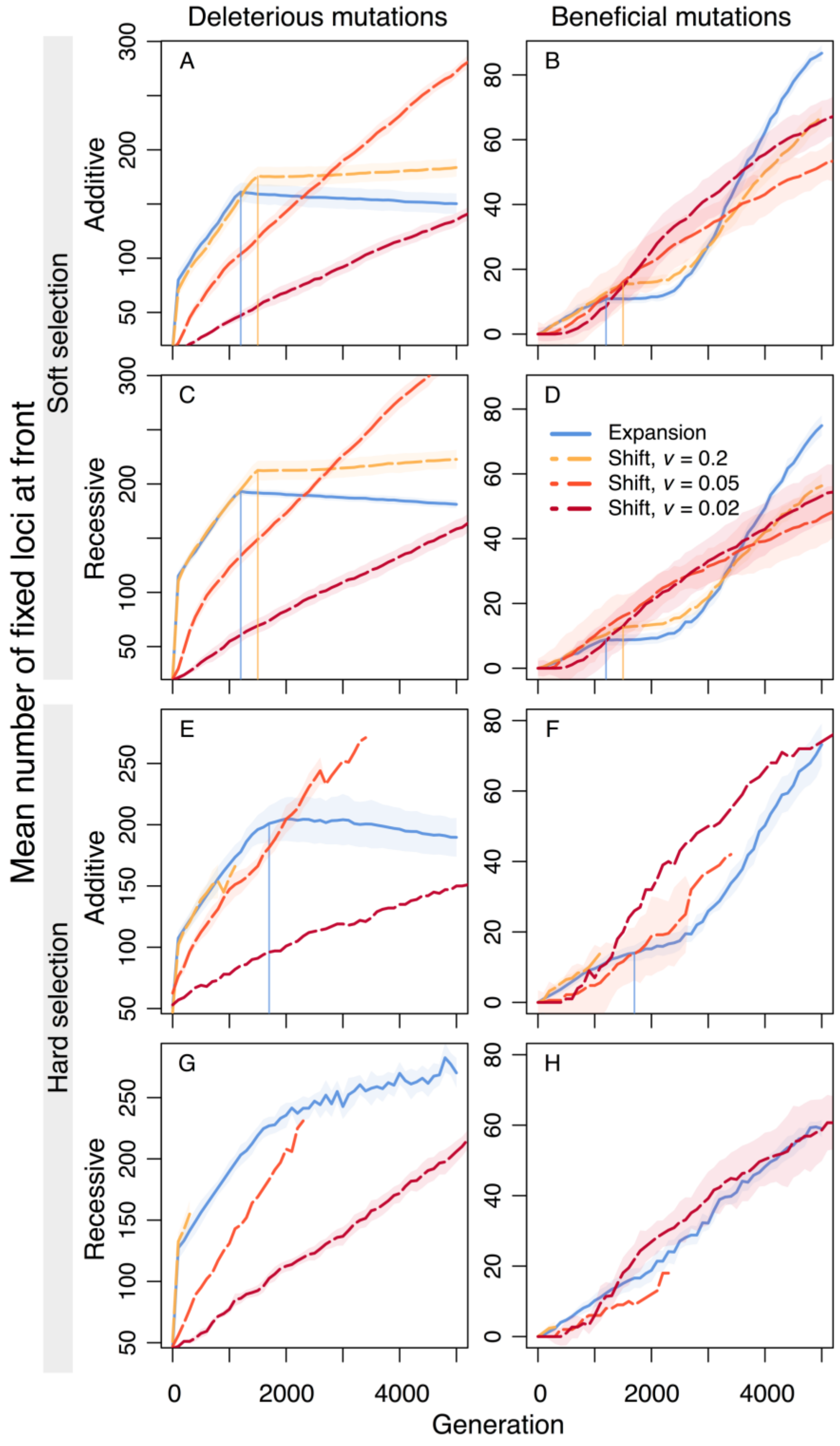

Supplement: S1 Fig — Fixation of deleterious (A, C, E, G) and beneficial (B, D, F, H) mutations at the expanding range front, under soft and hard selection on a 1-dimensional landscape. Vertical lines indicate when the landscape has been crossed and expansion is complete; extinction has occurred for lines that end abruptly. Shaded area indicates two standard errors over 10 replicates. (PDF) [file pgen.1007450.s003.pdf]
