# Supplementary material for: Mutation load dynamics during environmentally-driven range shifts
Source: PLoS Genet. 2018 Sep 28;14(9):e1007450. doi: 10.1371/journal.pgen.1007450 (PMC6179293; doi:10.1371/journal.pgen.1007450)

Figure S2. **Fitness change through time and space across parameter ranges.** The trade-off between mutations entering the population and selection acting upon these mutations combines to create the non-monotonic pattern of fitness loss seen across speeds of range shifts, as shown by our analytic model. The parameter set in the main text is seen in panels B, E, and H, where carrying capacity, K = 100 and migration rate, m = 0.1. The impact of beneficial mutations on fitness always decreases with faster speeds due to increasingly inefficient selection (panels A-C). Deleterious mutations impact fitness non-monotonically across speeds (panels A-C) because even though more mutations enter the system at slower speeds (more generations pass), selection is more efficient at removing them at slower speeds. Meanwhile at the fastest speeds drift is strongest, but fewer mutations are present (fewer generations for mutational input). Panels D-F show the combined impact of deleterious and beneficial mutations on fitness from panels A-C. With higher K and higher m, or extremely low m, the non-monotonic pattern of fitness loss per distance travelled is lost. Fitness loss per time (panels H-I) is always worse at faster speeds.

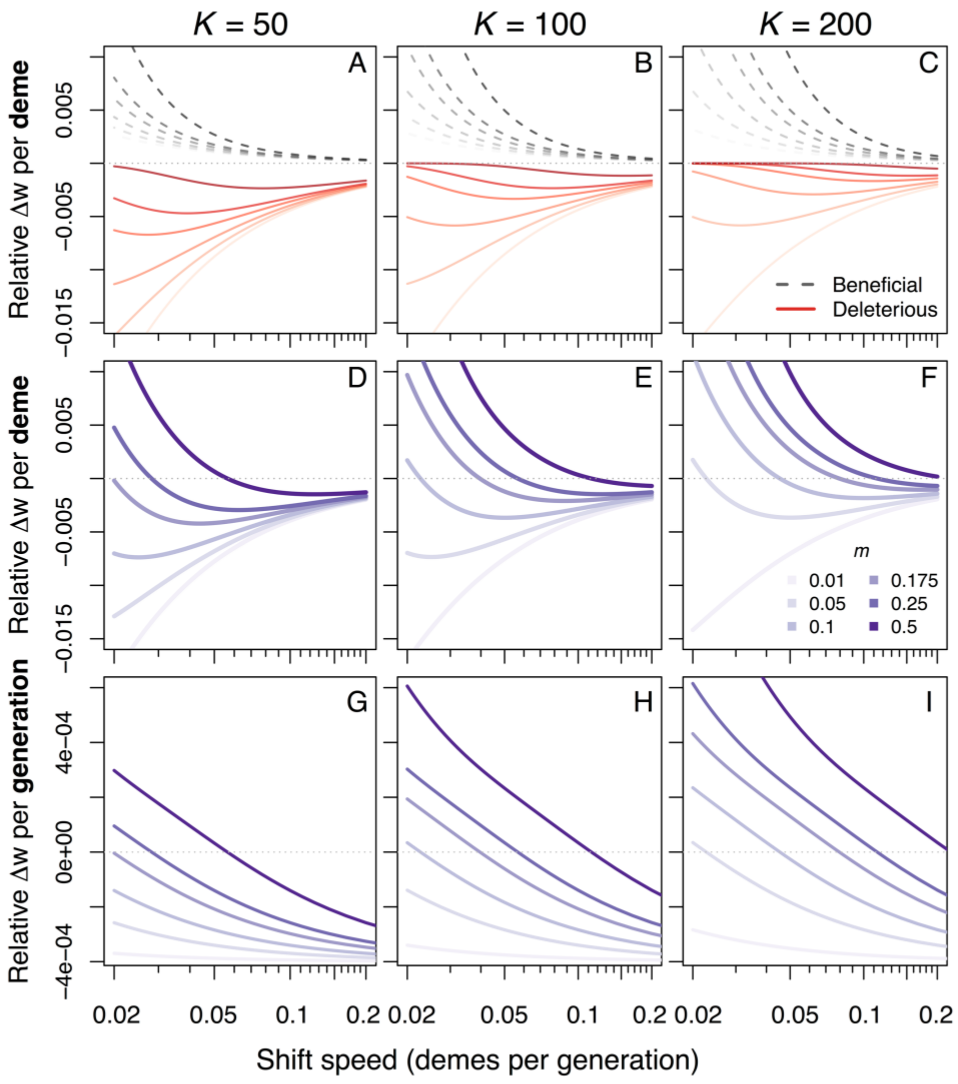

Supplement: S2 Fig — The trade-off between mutations entering the population and selection acting upon these mutations combines to create the non-monotonic pattern of fitness loss seen across speeds of range shifts, as shown by our analytic model. The parameter set in the main text is seen in panels B, E, and H, where carrying capacity, K = 100 and migration rate, m = 0.1. The impact of beneficial mutations on fitness always decreases with faster speeds due to increasingly inefficient selection (panels A-C). Deleterious mutations impact fitness non-monotonically across speeds (panels A-C) because even though more mutations enter the system at slower speeds (more generations pass), selection is more efficient at removing them at slower speeds. Meanwhile at the fastest speeds drift is strongest, but fewer mutations are present (fewer generations for mutational input). Panels D-F show the combined impact of deleterious and beneficial mutations on fitness from panels A-C. With higher K and higher m, or extremely low m, the non-monotonic pattern of fitness loss per distance travelled is lost. Fitness loss per time (panels H-I) is always worse at faster speeds. (PDF) [file pgen.1007450.s004.pdf]
