# Supplementary material for: Mutation load dynamics during environmentally-driven range shifts
Source: PLoS Genet. 2018 Sep 28;14(9):e1007450. doi: 10.1371/journal.pgen.1007450 (PMC6179293; doi:10.1371/journal.pgen.1007450)

Figure S3. Extinction due to reduced population growth. As fitness decreases at the front of a range shift due to expansion load (dashed lines), population growth decreases leading to increasingly small population sizes at the expanding front of range shifts (solid lines), under hard selection and with additive mutations. When fitness and thus population size reach a sufficiently low level, the population is no longer able to replace itself as fast as the pace of the shifting environment, resulting in extinction. This occurs more quickly with faster speeds of range shift, since fitness is lost faster through time and populations have less time to recover in size after colonizing new habitat. These analytic approximations qualitatively match our simulations (Figure 3A).

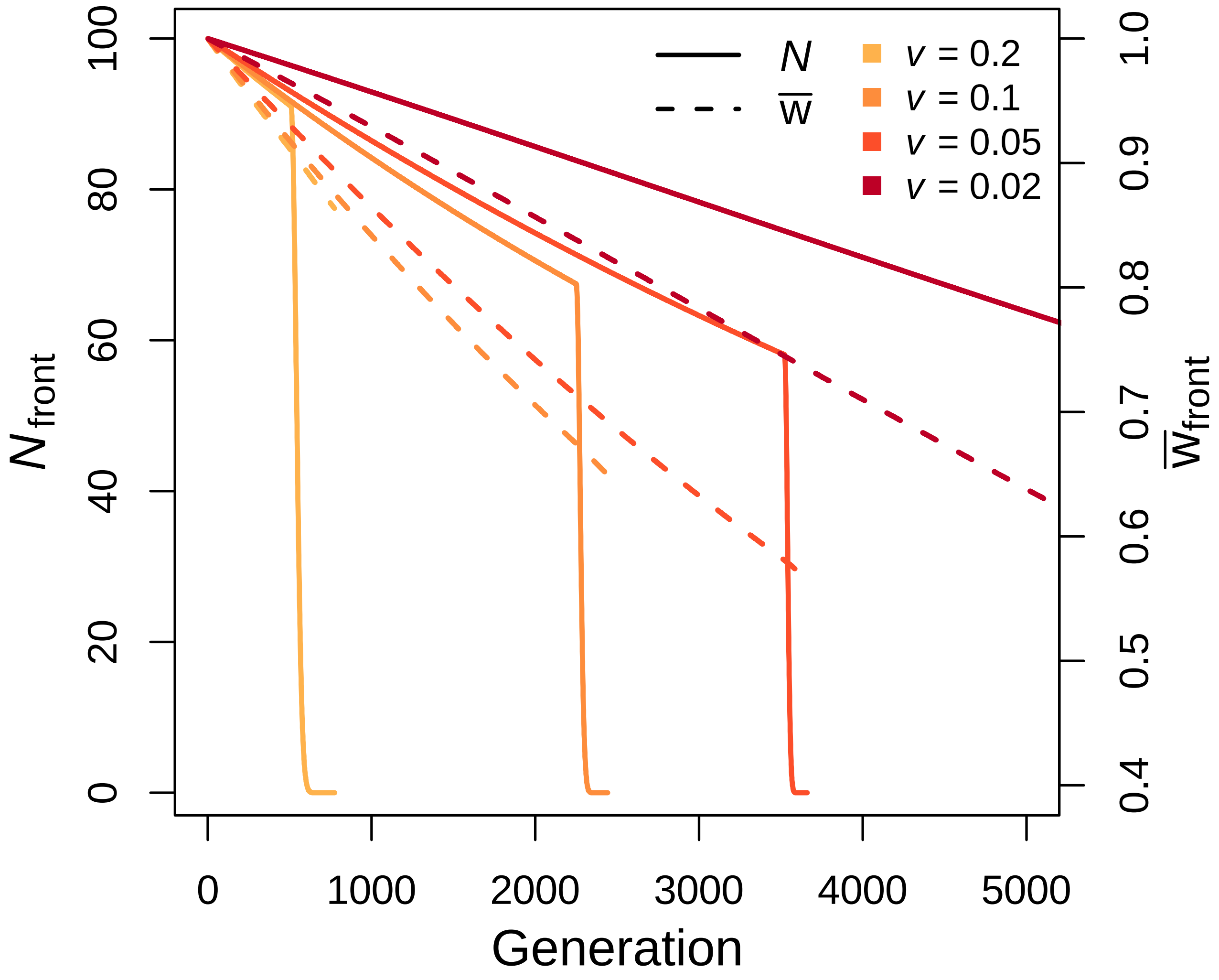

Supplement: S3 Fig — As fitness decreases at the front of a range shift due to expansion load (dashed lines), population growth decreases leading to increasingly small population sizes at the expanding front of range shifts (solid lines), under hard selection and with additive mutations. When fitness and thus population size reach a sufficiently low level, the population is no longer able to replace itself as fast as the pace of the shifting environment, resulting in extinction. This occurs more quickly with faster speeds of range shift, since fitness is lost faster through time and populations have less time to recover in size after colonizing new habitat. These analytic approximations qualitatively match our simulations (Fig 3A). (PDF) [file pgen.1007450.s005.pdf]
