# Supplementary material for: Mutation load dynamics during environmentally-driven range shifts
Source: PLoS Genet. 2018 Sep 28;14(9):e1007450. doi: 10.1371/journal.pgen.1007450 (PMC6179293; doi:10.1371/journal.pgen.1007450)

Figure S4. Neutral genetic diversity through time. Neutral diversity over 1000 neutral loci during and after range expansion and shifts at both the expanding edge and in the core (which is calculated as the rear-most deme in range shifts, i.e. the receding edge). Shading indicates 95% confidence intervals over 20 replicates (10 replicates under additive model for selected loci, 10 replicates under recessive model for selected loci). Vertical lines in the left panel indicate when the landscape is crossed and expansion is complete. Slower shifts do not cross the landscape within 5,000 generations. Four various speeds of range shifts are compared.

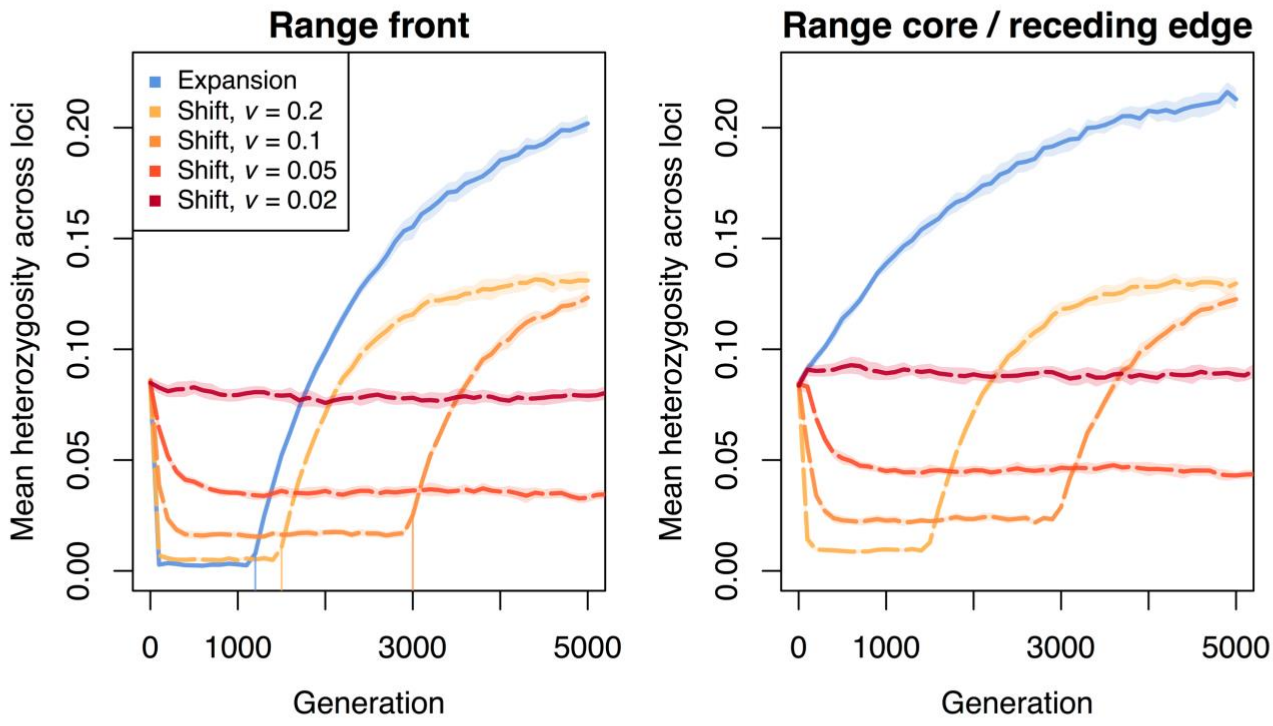

Supplement: S4 Fig — Neutral diversity over 1000 neutral loci during and after range expansion and shifts at both the expanding edge and in the core (which is calculated as the rear-most deme in range shifts, i.e. the receding edge). Shading indicates 95% confidence intervals over 20 replicates (10 replicates under additive model for selected loci, 10 replicates under recessive model for selected loci). Vertical lines in the left panel indicate when the landscape is crossed and expansion is complete. Slower shifts do not cross the landscape within 5,000 generations. Four various speeds of range shifts are compared. (PDF) [file pgen.1007450.s006.pdf]
