# Supplementary material for: Mutation load dynamics during environmentally-driven range shifts
Source: PLoS Genet. 2018 Sep 28;14(9):e1007450. doi: 10.1371/journal.pgen.1007450 (PMC6179293; doi:10.1371/journal.pgen.1007450)

Supplementary 2-dimensional landscape analyses

We additionally modelled 5-deme-wide, two-dimensional landscapes with migration to the four neighboring demes (m = 0.1), as these scenarios should be more biologically realistic. We compared four cases by varying both the carrying capacity (K) of the demes and the width of the landscape (1- or 2-dimensional): our standard 1-D model with K = 100, a 2-D model with K = 20 per deme (implying a total front size of 100), a 1-D model with K = 500 per deme, and a 2-D model with K = 100 per deme (implying a total front size of 500). This allows us to disentangle per-deme versus per-front differences in population sizes by creating scenarios with approximately the same number of individuals on the expansion front across models. The effective population sizes at the range front will differ across these scenarios due to population sub-structuring in 2-D.

The main driver of differences among these results is the population size at the expanding front. When population size at the front is approximately 500 (1-D K = 500 or 2-D K = 100) populations recover to a higher level of fitness at a faster rate than for smaller population sizes (1-D K = 100 or 2-D K = 20). Substructure likely does not improve fitness for the K = 20 scenario as drift within each subpopulation will still be strong at this small size. This is especially apparent during a range shift (Figure S5B) where the smaller population sizes in both 1- and 2-D suffer from more fitness loss while expanding and recover fitness at much lower rates than the larger populations. The most notable impact of a 2-dimensional landscape is during the recovery after a range shift under the recessive model (Figure S5D), where the 2-D K = 100 model shows the greatest recovery from equivalent losses in fitness during the shift. Under hard selection, we also observe extinctions during range shifts in 2-D simulations, and qualitative differences between the additive and recessive models are similar to those described under soft selection (Figure S6).

Figure S5. Soft selection 2-dimensional range expansions and shifts. Range expansions (panels A and C) and shifts (v = 0.2; panels B and D) for additive and recessive mutational models in two dimensions are compared for cases where either the population size across the 5-deme-wide front is equivalent to population size in the 1-deme-wide front (2D K = 20 vs. 1D K =100 and 2D K = 100 vs. 1D K = 500), or alternatively where the per-deme carrying capacity, K, is held constant across comparisons (2D K = 100 vs. 1D K = 100). Shaded regions show two standard errors calculated over ten replicate simulations. Vertical lines indicate when the landscape has been crossed and expansion is complete

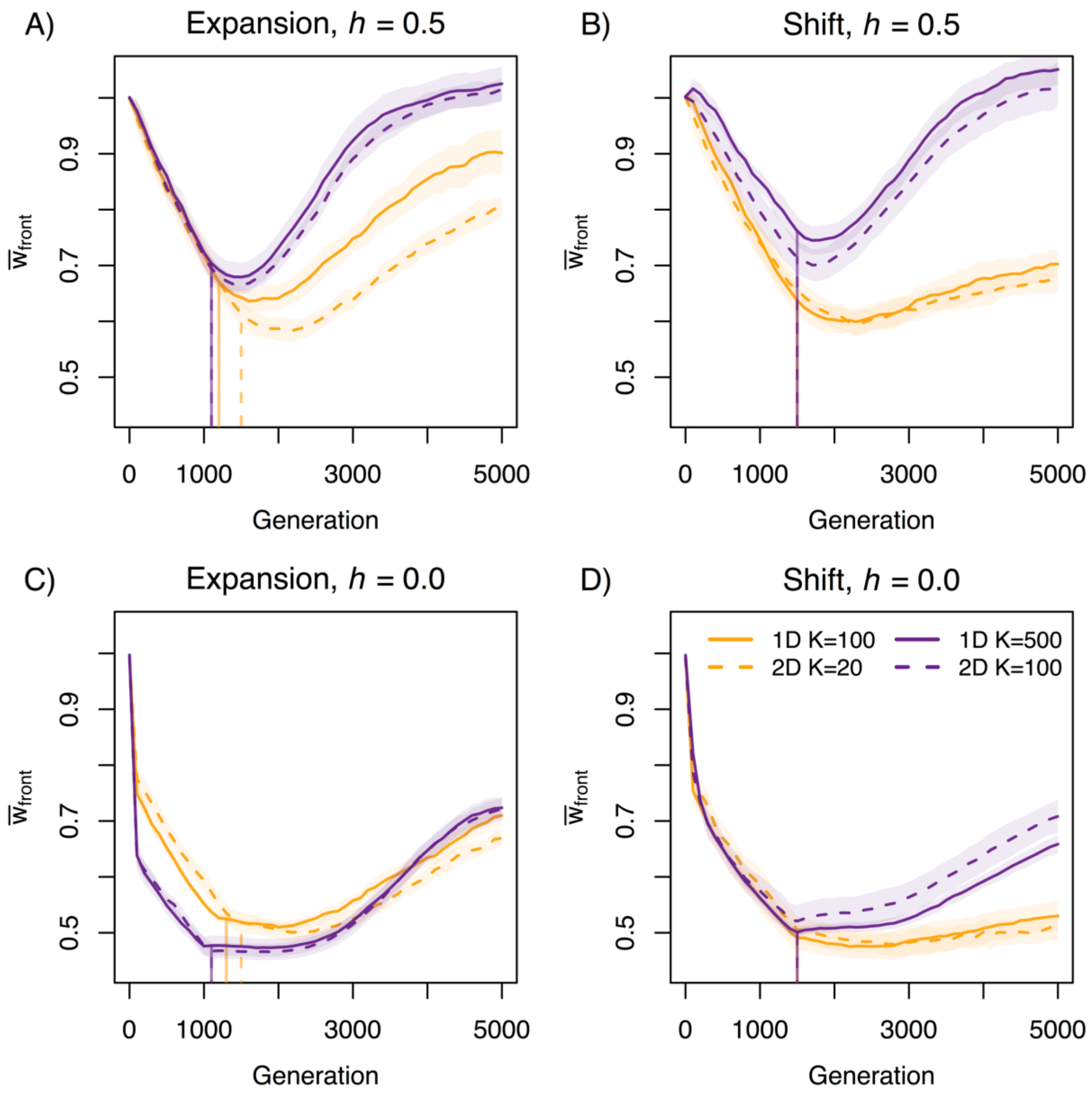

Supplement: S5 Fig — Range expansions (panels A and C) and shifts (v = 0.2; panels B and D) for additive and recessive mutational models in two dimensions are compared for cases where either the population size across the 5-deme-wide front is equivalent to population size in the 1-deme-wide front (2D K = 20 vs. 1D K = 100 and 2D K = 100 vs. 1D K = 500), or alternatively where the per-deme carrying capacity, K, is held constant across comparisons (2D K = 100 vs. 1D K = 100). Shaded regions show two standard errors calculated over ten replicate simulations. Vertical lines indicate when the landscape has been crossed and expansion is complete. (PDF) [file pgen.1007450.s007.pdf]
