# Supplementary material for: Mutation load dynamics during environmentally-driven range shifts
Source: PLoS Genet. 2018 Sep 28;14(9):e1007450. doi: 10.1371/journal.pgen.1007450 (PMC6179293; doi:10.1371/journal.pgen.1007450)

Figure S6. Hard selection 2-dimensional range expansions and shifts. Results for fitness change of 2-D versus 1-D simulations under hard selection. Results are shown for range expansions (panels A and C) and shifts (*v* = 0.2; panels B and D) for additive and recessive mutational models, respectively. Shaded regions indicate two standard errors over 10 replicates. Vertical lines indicate when the landscape has been crossed and expansion is complete. Absence of a line indicates extinction.

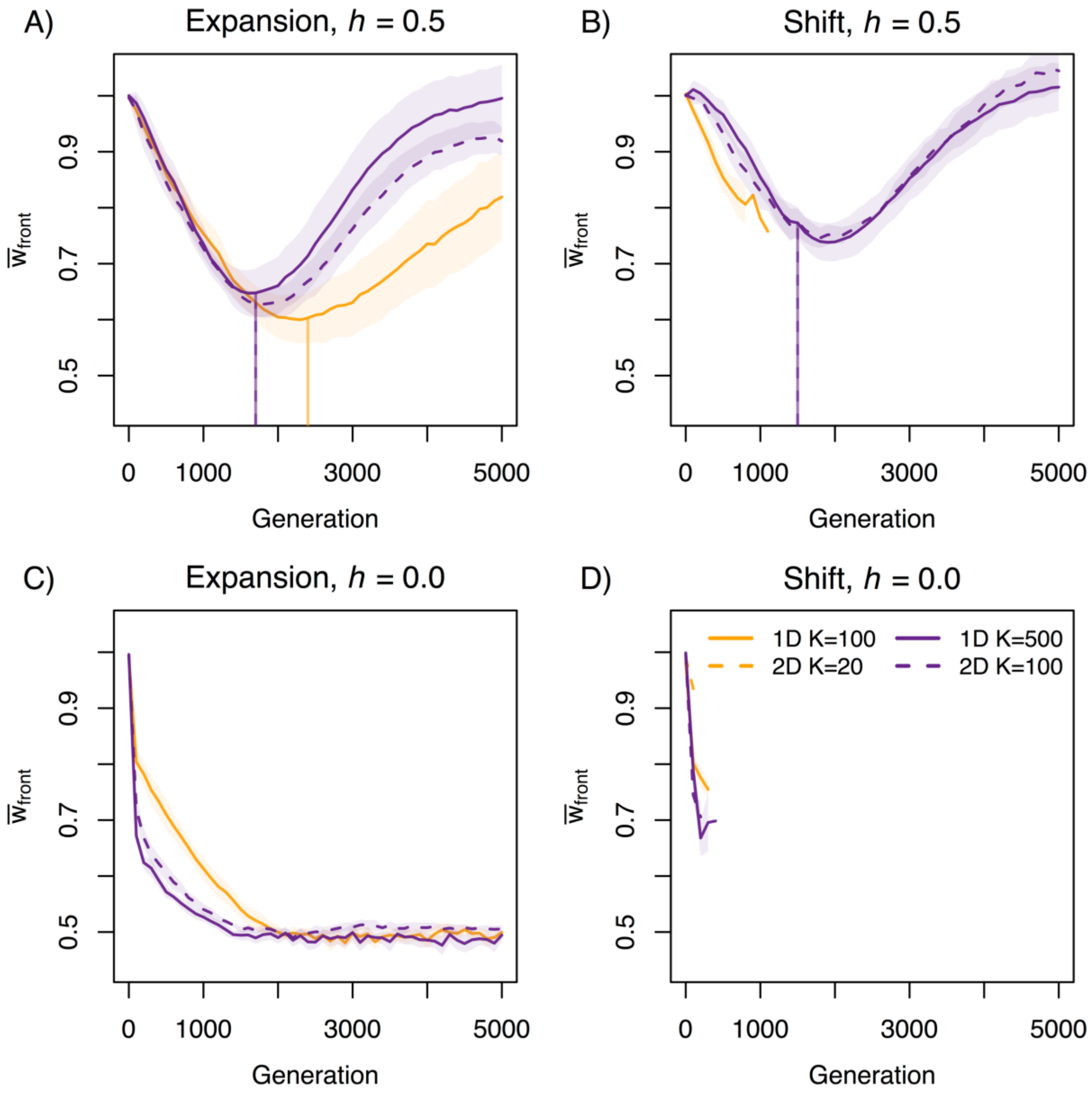

Supplement: S6 Fig — Results for fitness change of 2-D versus 1-D simulations under hard selection. Results are shown for range expansions (panels A and C) and shifts (v = 0.2; panels B and D) for additive and recessive mutational models, respectively. Shaded regions indicate two standard errors over 10 replicates. Vertical lines indicate when the landscape has been crossed and expansion is complete. Absence of a line indicates extinction. (PDF) [file pgen.1007450.s008.pdf]
