# Supplementary material for: Mutation load dynamics during environmentally-driven range shifts
Source: PLoS Genet. 2018 Sep 28;14(9):e1007450. doi: 10.1371/journal.pgen.1007450 (PMC6179293; doi:10.1371/journal.pgen.1007450)

Figure S7. Recovery due to beneficial mutations. Ridgeline plots of allele frequency change through time across the exponential distribution of fitness effect sizes, described in the Methods. Locus allele frequencies have been binned into equal-sized bins of 10 loci each, across the 900 deleterious and 100 beneficial loci, making each line represent 100 bins across the range of the selection coefficient, *s*, rather than 1000 loci. Each individual line across the *y*-axis is a sampled time point, with the start of the simulation being the top- (or back-) most line. Allele frequencies range from 0 to 1 on the *z*-axis. Panel A shows results for an expansion where $h = 0.3$, panel B a range shift where $h = 0.3$, panel C an expansion with a trade-off between *h*-*s*, and panel D a range shift with a trade-off between *h*-*s*.

A) Full expansion, $h = 0.3$

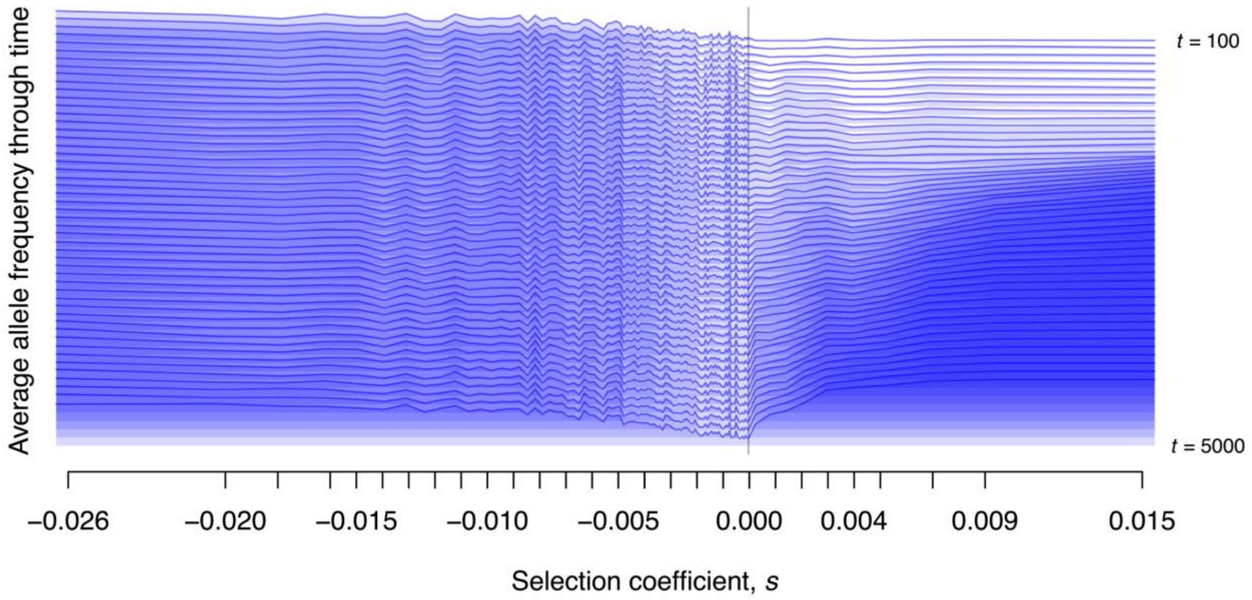

B) Range shift $v = 0.2$, $h = 0.3$

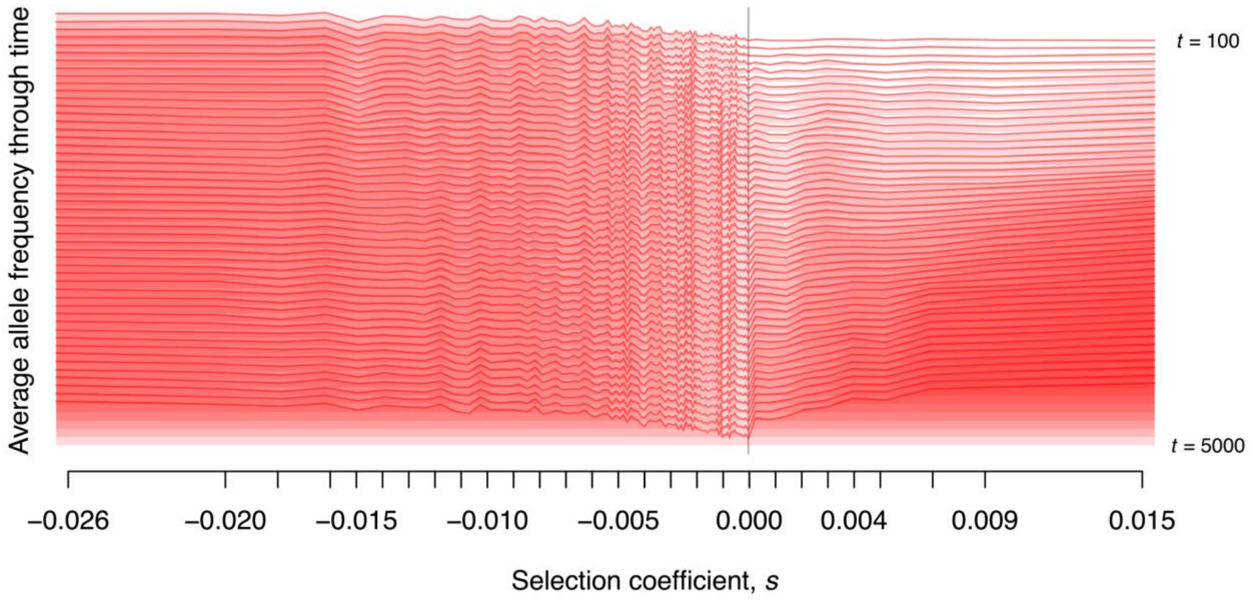

C) Full expansion, *h*-*s* trade-off

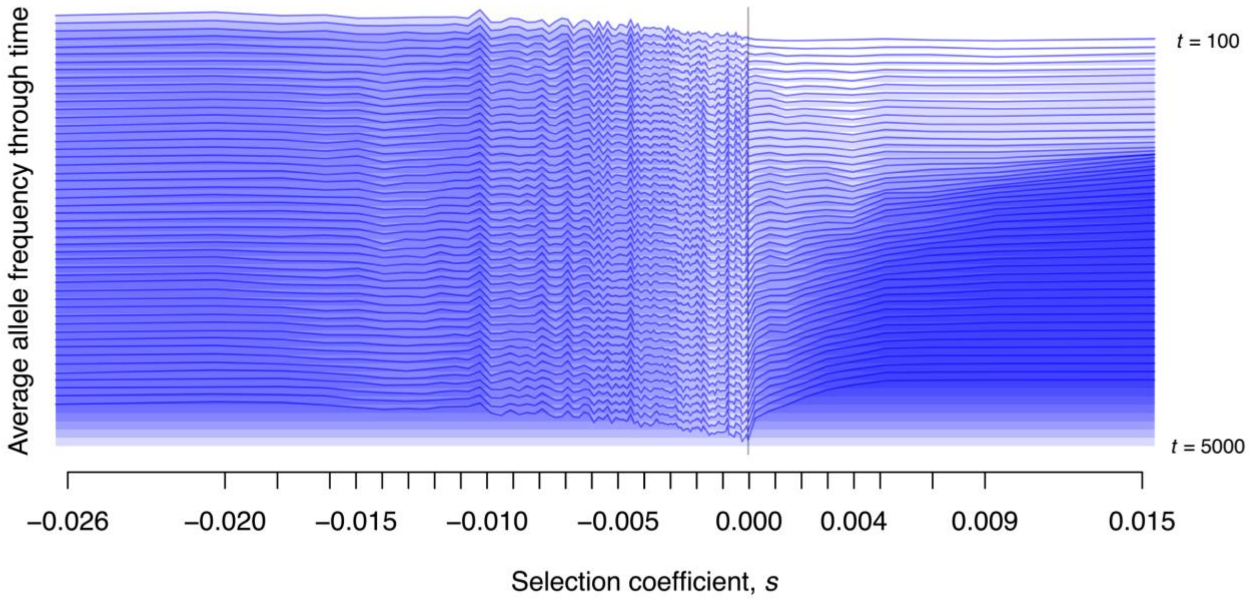

D) Range shift, $v = 0.2$, *h*-*s* trade-off

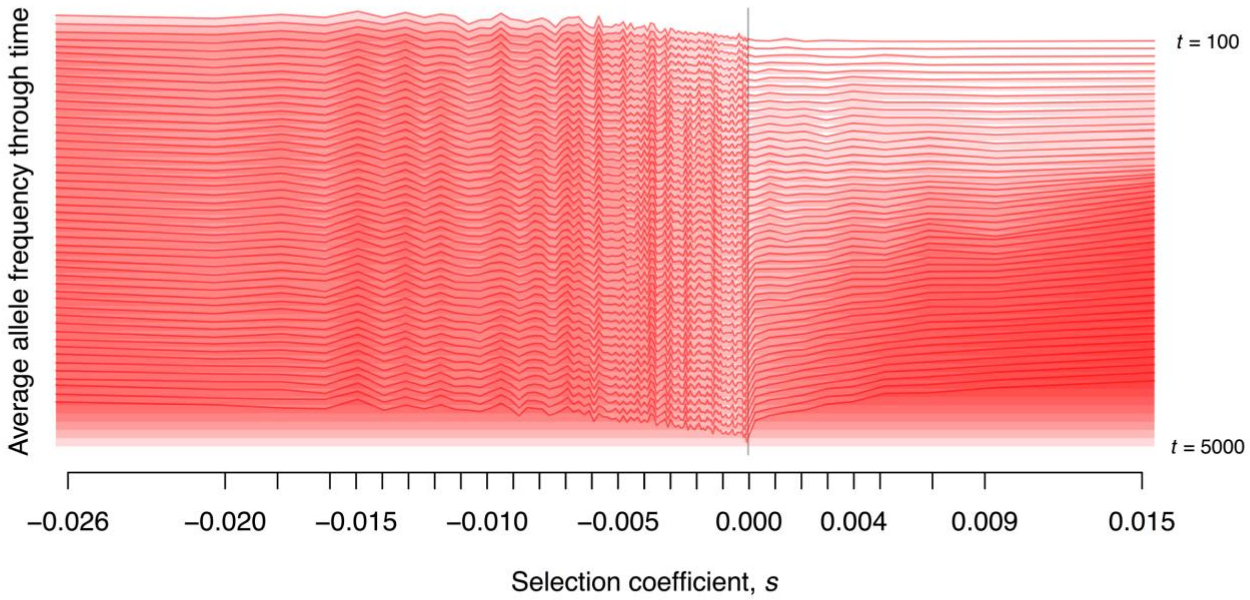

Supplement: S7 Fig — Ridgeline plots of allele frequency change through time across the exponential distribution of fitness effect sizes, described in the Methods. Locus allele frequencies have been binned into equal-sized bins of 10 loci each, across the 900 deleterious and 100 beneficial loci, making each line represent 100 bins across the range of the selection coefficient, s, rather than 1000 loci. Each individual line across the y-axis is a sampled time point, with the start of the simulation being the top- (or back-) most line. Allele frequencies range from 0 to 1 on the z-axis. Panel A shows results for an expansion where h = 0.3, panel B a range shift where h = 0.3, panel C an expansion with a trade-off between h-s, and panel D a range shift with a trade-off between h-s. (PDF) [file pgen.1007450.s009.pdf]
