# Supplementary material for: Mutation load dynamics during environmentally-driven range shifts
Source: PLoS Genet. 2018 Sep 28;14(9):e1007450. doi: 10.1371/journal.pgen.1007450 (PMC6179293; doi:10.1371/journal.pgen.1007450)

Figure S8. Mutation fixation under various mutation models. Deleterious (A) and beneficial (B) mutation fixation at the range edge across range expansions and range shifts, over varying mutational models of *h* and *s* as indicated in the figure legend.

A)

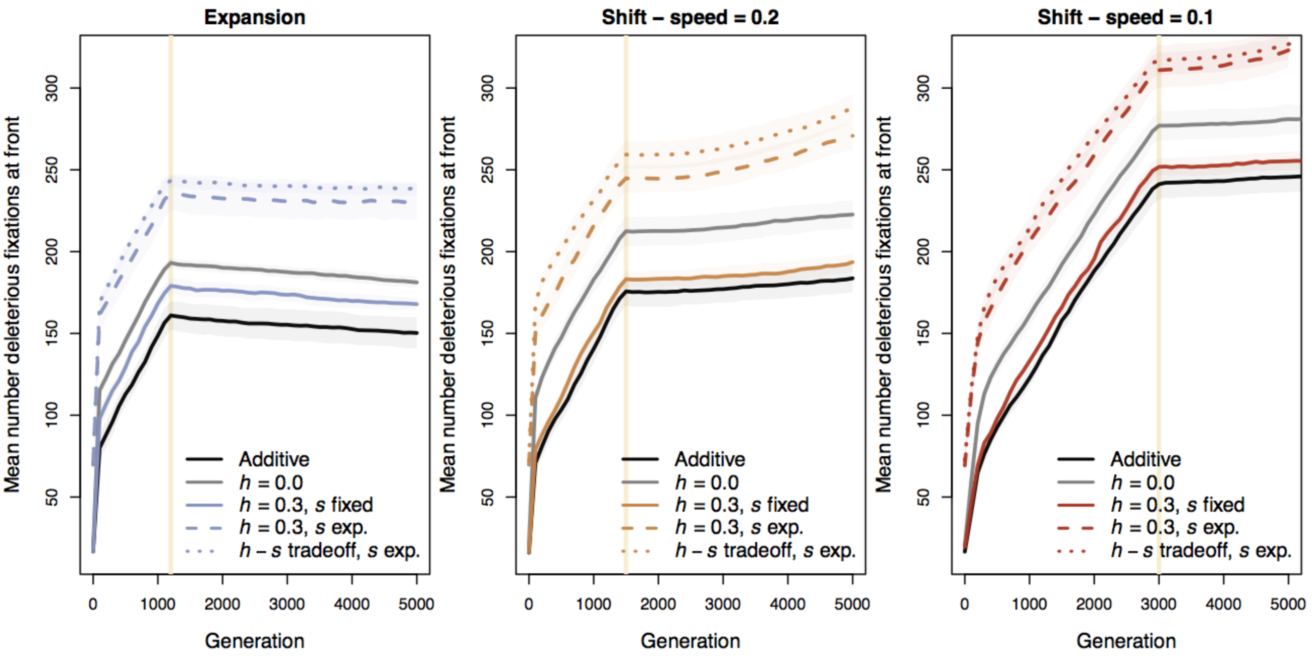

B)

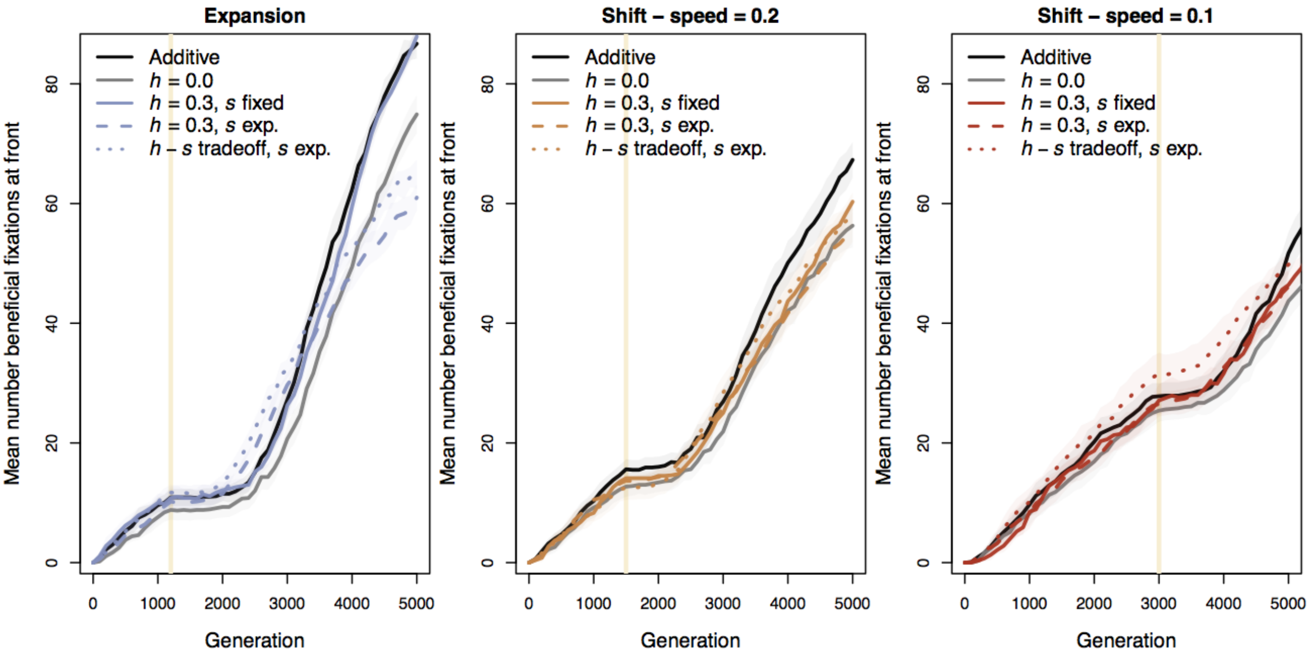

Supplement: S8 Fig — Deleterious (A) and beneficial (B) mutation fixation at the range edge across range expansions and range shifts, over varying mutational models of h and s as indicated in the figure legend. (PDF) [file pgen.1007450.s010.pdf]
