# Supplementary material for: Mutation load dynamics during environmentally-driven range shifts
Source: PLoS Genet. 2018 Sep 28;14(9):e1007450. doi: 10.1371/journal.pgen.1007450 (PMC6179293; doi:10.1371/journal.pgen.1007450)

S9 Fig. Fitness trajectories in the absence of recombination. Trajectories of mean fitness loss over time (panels A and B) and space (panels C and D) for additive and recessive models, respectively, at the expanding front under soft selection show similar fitness loss between cases of free recombination ($r = 0.5$) and no recombination ($r = 0.0$) during spread. However, this extreme case of the absence of recombination shows that recovery does not occur, and instead fitness is continually lost after crossing the landscape since beneficial and deleterious mutations are fully linked and purging is prevented. Under the recessive model with no recombination, more noise is seen in fitness at the front, due to high-fitness migrants from the core invading the front, as shown by [28]. Vertical lines indicate when the population reaches the end of the 1x300 deme landscape and expansion is complete. Shaded regions show two standard errors calculated over ten replicate simulations.

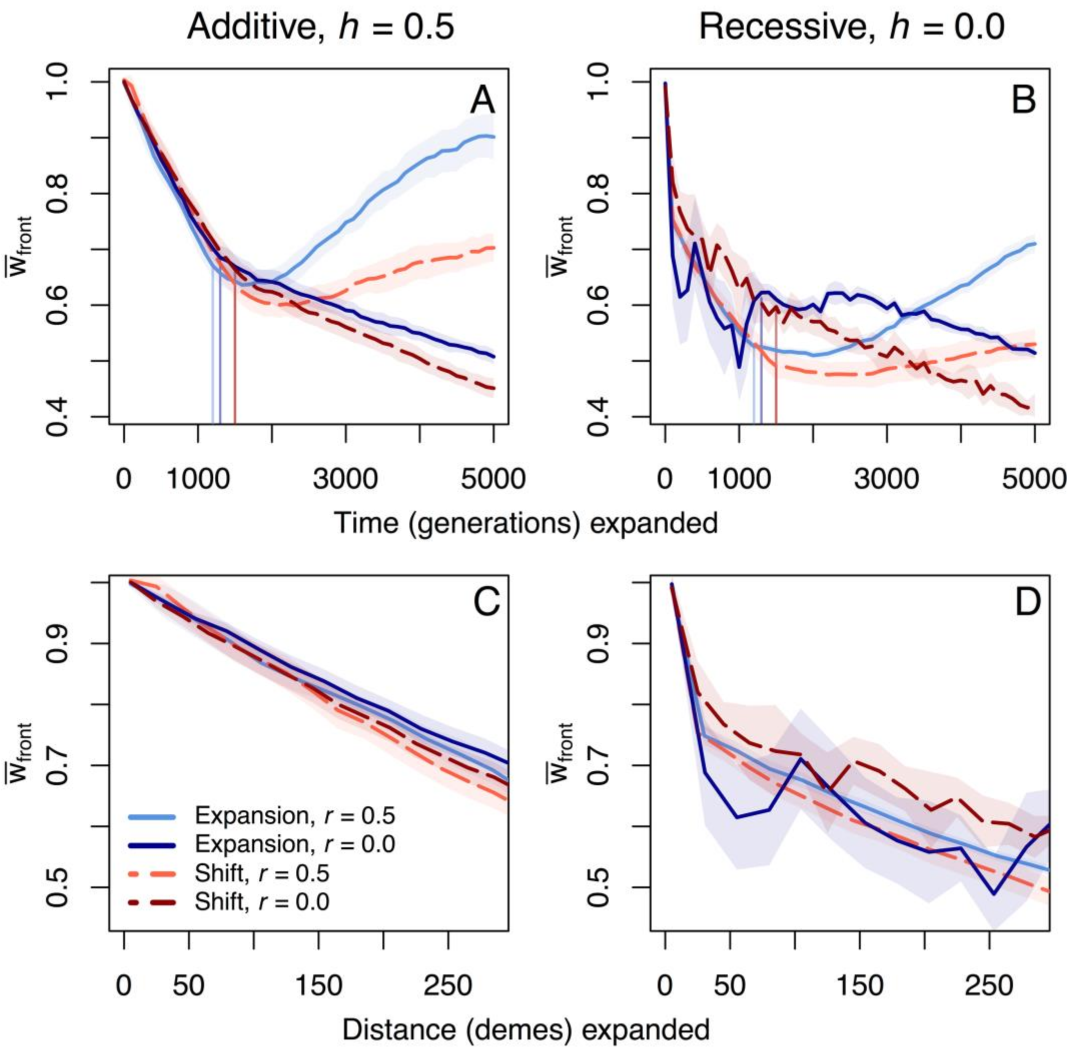

Supplement: S9 Fig — Trajectories of mean fitness loss over time (panels A and B) and space (panels C and D) for additive and recessive models, respectively, at the expanding front under soft selection show similar fitness loss between cases of free recombination (r = 0.5) and no recombination (r = 0.0) during spread. However, this extreme case of the absence of recombination shows that recovery does not occur, and instead fitness is continually lost after crossing the landscape since beneficial and deleterious mutations are fully linked and purging is prevented. Under the recessive model with no recombination, more noise is seen in fitness at the front, due to high-fitness migrants from the core invading the front, as shown by [28]. Vertical lines indicate when the population reaches the end of the 1x300 deme landscape and expansion is complete. Shaded regions show two standard errors calculated over ten replicate simulations. (PDF) [file pgen.1007450.s011.pdf]
