# Supplementary material for: Mutation load dynamics during environmentally-driven range shifts
Source: PLoS Genet. 2018 Sep 28;14(9):e1007450. doi: 10.1371/journal.pgen.1007450 (PMC6179293; doi:10.1371/journal.pgen.1007450)

Figure S10. *h-s* tradeoff. The *h-s* relationship modelled for deleterious mutations under the *h-s* trade-off scenarios shown in Results Figure 4. (see Methods for description)

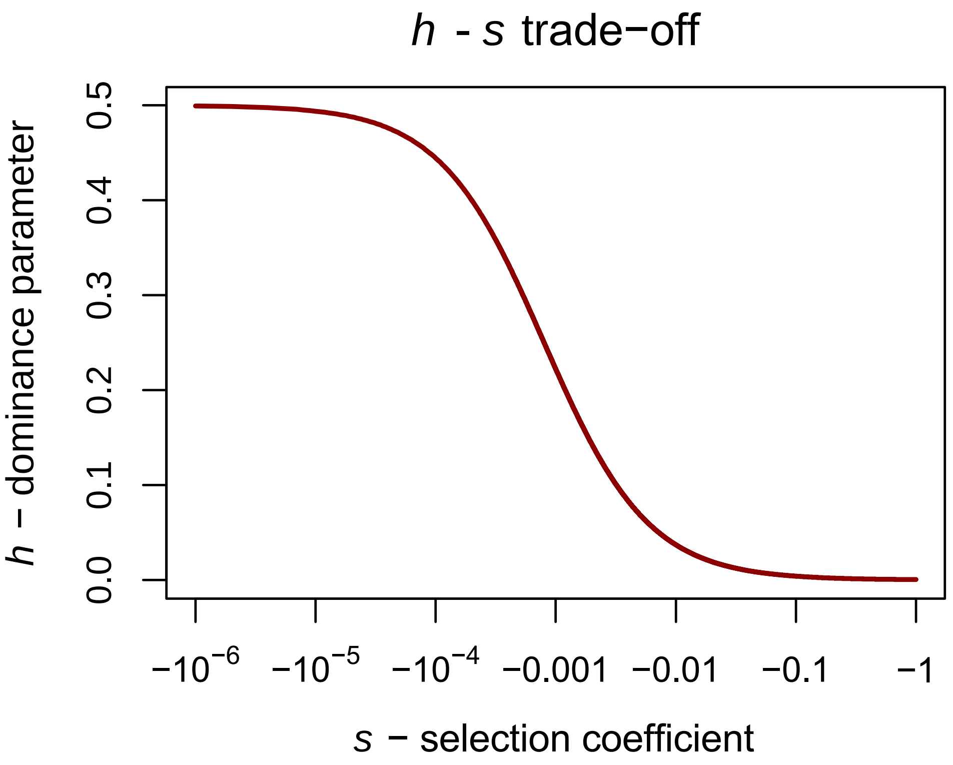

Supplement: S10 Fig — The h-s relationship modelled for deleterious mutations under the h-s trade-off scenarios shown in Results Fig 4. (see Methods for description). (PDF) [file pgen.1007450.s012.pdf]
