# Supplementary material for: Mutation load dynamics during environmentally-driven range shifts
Source: PLoS Genet. 2018 Sep 28;14(9):e1007450. doi: 10.1371/journal.pgen.1007450 (PMC6179293; doi:10.1371/journal.pgen.1007450)

Figure S11. Equilibrium expansion speeds. Results under simulations with hard selection for sufficiently slow speeds of range shift show that fitness is on average neither gained or lost at the expanding front, until mutations begin to saturate between generations 2,000 - 3,000. Under an additive mutation model, this speed is realized at 0.017 demes per generation (v = 1/60) and under a recessive model at 0.012 demes per generation (v = 1/84).

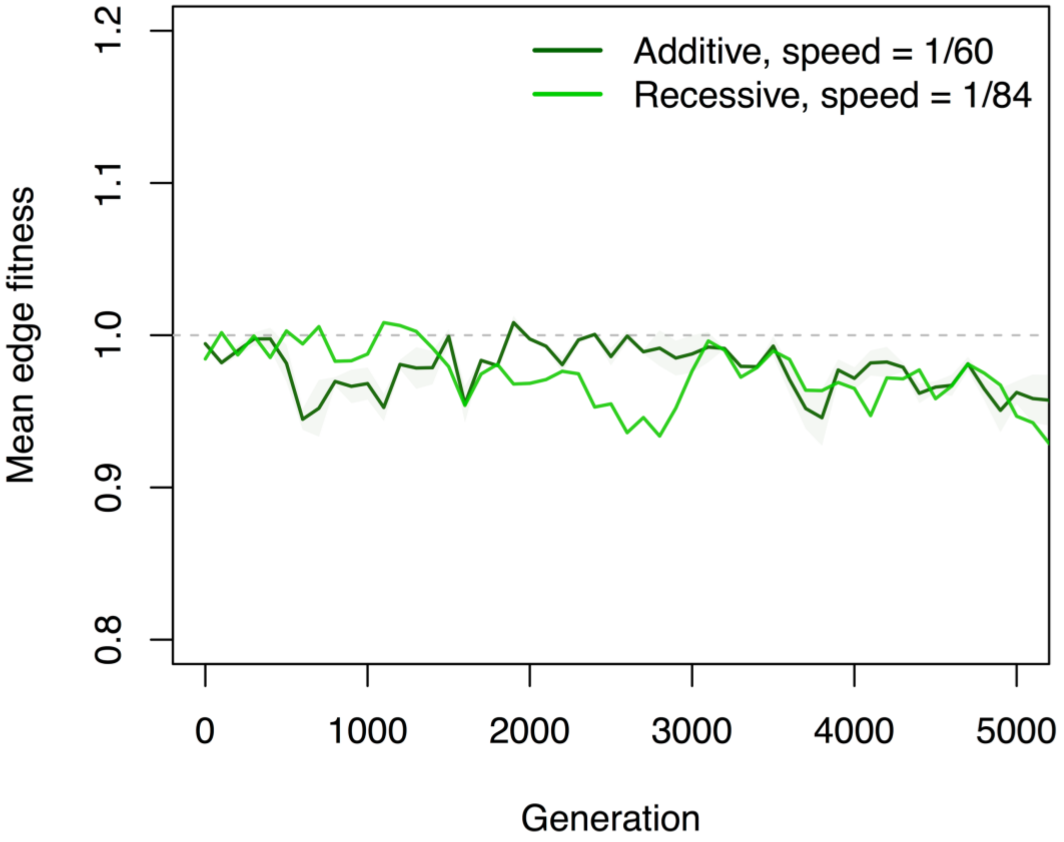

Supplement: S11 Fig — Results under simulations with hard selection for sufficiently slow speeds of range shift show that fitness is on average neither gained or lost at the expanding front, until mutations begin to saturate between generations 2,000–3,000. Under an additive mutation model, this speed is realized at 0.017 demes per generation (v = 1/60) and under a recessive model at 0.012 demes per generation (v = 1/84). (PDF) [file pgen.1007450.s013.pdf]
