# Supplementary material for: Mutation load dynamics during environmentally-driven range shifts
Source: PLoS Genet. 2018 Sep 28;14(9):e1007450. doi: 10.1371/journal.pgen.1007450 (PMC6179293; doi:10.1371/journal.pgen.1007450)

Figure S12. Fitness loss per time and space under soft selection. Trajectories of mean fitness loss over time (panels A and B) and space (panels C and D) for additive and recessive models, respectively, at the expanding front under soft selection are shown over a longer timescale than Fig 1 to show the full loss of fitness and recovery trajectory for the two cases of slower range shifts. Vertical lines indicate when the population reaches the end of the 1x300 deme landscape and expansion is complete. Shaded regions show two standard errors calculated over ten replicate simulations. All lines in panels A and B end simply because the time course of the simulations has completed (no extinction occurs under soft selection).

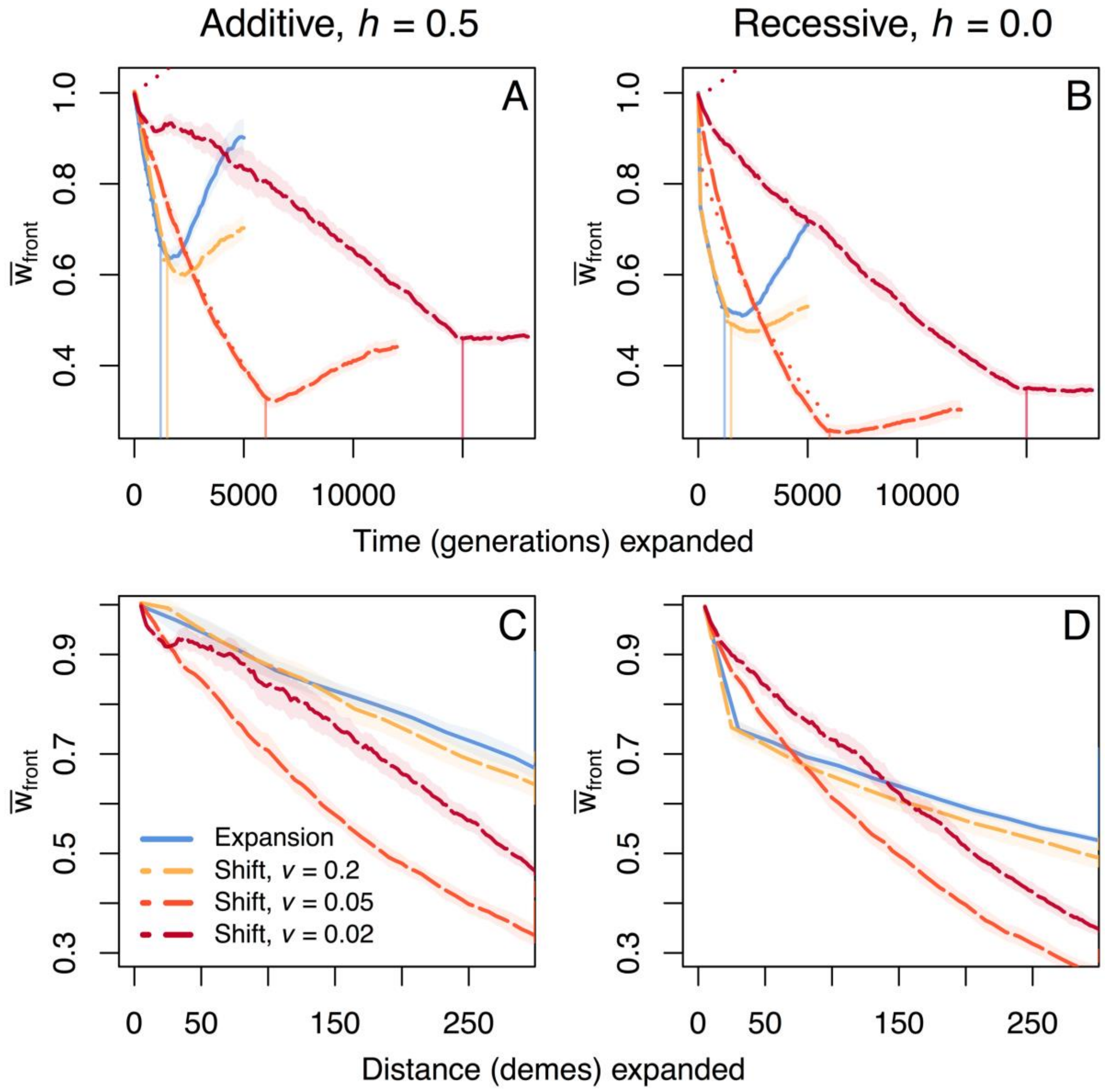

Supplement: S12 Fig — Trajectories of mean fitness loss over time (panels A and B) and space (panels C and D) for additive and recessive models, respectively, at the expanding front under soft selection are shown over a longer timescale than Fig 1 to show the full loss of fitness and recovery trajectory for the two cases of slower range shifts. Vertical lines indicate when the population reaches the end of the 1x300 deme landscape and expansion is complete. Shaded regions show two standard errors calculated over ten replicate simulations. All lines in panels A and B end simply because the time course of the simulations has completed (no extinction occurs under soft selection). (PDF) [file pgen.1007450.s014.pdf]
